# Effect of mirror therapy combined with transcranial direct current stimulation on upper limb motor function in stroke patients: A meta-analysis

**DOI:** 10.1097/MD.0000000000050057

**Published:** 2026-07-31

**Authors:** Beibei Zong, Chun Zhang, Qingsha Zhang

**Affiliations:** a Department of Rehabilitation Medicine, Zibo Central Hospital, Zibo, Shandong, China.

**Keywords:** meta-analysis, mirror therapy, motor function, stroke, transcranial direct current stimulation, upper limb

## Abstract

**Background:**

Upper limb motor impairment is a major contributor to long-term disability after stroke. Mirror therapy (MT) promotes motor relearning through visuomotor feedback, whereas transcranial direct current stimulation (tDCS) may facilitate neuroplasticity by modulating cortical excitability. We conducted a systematic review and meta-analysis to evaluate whether MT combined with tDCS provides additional benefits for poststroke upper limb rehabilitation.

**Methods:**

We searched PubMed, Embase, Web of Science, the Cochrane Library, CNKI, and Wanfang for randomized controlled trials (RCTs) published from 2015 to November 2025. Eligible studies enrolled adults with stroke-related upper limb motor deficits and compared MT + tDCS with MT alone or tDCS alone.

**Results:**

Twelve RCTs involving 1024 participants were included. For Fugl-Meyer Assessment for Upper Extremity, MT + tDCS showed superior improvement versus MT alone (3 studies; I-squared statistic (*I*^2^) = 21.9%; MD = 12.65, 95% confidence interval [CI] 10.07–15.23) and versus tDCS alone (7 studies; *I*^2^ = 93.3%; MD = 7.03, 95% CI 3.76–10.31). For activities of daily living, the effect on Modified Barthel Index versus MT alone was highly heterogeneous and imprecise (2 studies; *I*^2^ = 94.7%; MD = 24.19, 95% CI − 144.99 to 193.38), whereas MT + tDCS significantly improved Modified Barthel Index versus tDCS alone (5 studies; *I*^2^ = 42.7%; MD = 9.29, 95% CI 6.36–12.22). For hand function, MT + tDCS improved Wolf Motor Function Test versus tDCS alone (3 studies; *I*^2^ = 80.3%; MD = 4.90, 95% CI 0.59–9.22). Reported adverse events were generally mild and transient.

**Conclusion:**

MT combined with tDCS appears to provide additional benefits for poststroke upper limb motor recovery, with consistent improvements in Fugl-Meyer Assessment for Upper Extremity and favorable effects on daily functioning and hand performance in key comparisons. However, heterogeneity across protocols and imprecision in some outcomes limit certainty. Larger, rigorously designed RCTs with standardized MT dosing and tDCS parameters and longer follow-up are warranted.

## 1. Introduction

With population aging and lifestyle changes, the global burden of stroke continues to rise. Stroke remains one of the leading causes of long-term disability worldwide and a major cause of death in adults.^[1]^ The high rate of poststroke disability substantially compromises quality of life, and many survivors develop persistent motor deficits, including limb weakness, restricted joint mobility, impaired balance, and poor coordination.^[2]^ Upper limb dysfunction is particularly common and clinically important because it directly limits activities of daily living (ADLs) such as eating, dressing, and personal hygiene, thereby reducing independence and social participation. Although conventional rehabilitation has advanced considerably, a substantial proportion of patients still experience residual arm–hand impairment, especially in the chronic stage, highlighting the need for adjunctive interventions that specifically target neuroplasticity.^[3]^

Mirror therapy (MT) is a widely used and cost-effective rehabilitation approach that creates a visual illusion of normal movement of the affected limb through mirror visual feedback.^[4]^By engaging motor-related neural circuits via action observation–execution coupling and the mirror neuron system, MT may enhance sensorimotor integration and facilitate motor relearning, making it a valuable component of poststroke upper limb rehabilitation.^[5]^ Nevertheless, MT protocols vary across studies in terms of task selection, progression rules, training frequency, and overall treatment dose. In practice and in many trials, MT typically consists of structured, repetitive unilateral or bilateral task practice, with sessions lasting approximately 30 minutes and delivered multiple times per week over several weeks: parameters that may influence treatment effects and contribute to between-study variability.^[6]^

Transcranial direct current stimulation (tDCS) is a noninvasive brain stimulation technique that delivers a weak direct current (commonly 1–2 mA) through scalp electrodes to modulate cortical excitability. In general, anodal stimulation increases, whereas cathodal stimulation decreases excitability in the targeted cortical region.^[7]^ In stroke rehabilitation, tDCS is frequently applied over the primary motor cortex (M1) to promote adaptive neuroplasticity and mitigate maladaptive interhemispheric imbalance. Importantly, tDCS is often combined with behavioral training, based on the “priming” concept that neuromodulation may increase cortical responsiveness to practice and thereby amplify training-dependent functional gains.^[8]^

In recent years, combining tDCS with MT has attracted growing interest as a “central–peripheral” neuromodulation strategy: MT provides behaviorally driven action observation and sensorimotor training, while tDCS modulates cortical excitability to optimize the neural milieu for learning and reorganization. Emerging evidence suggests that MT plus tDCS may yield greater improvements in upper limb motor function and ADLs than either intervention alone.^[9]^However, trial findings remain variable, likely due to differences in stimulation parameters, MT dose, intervention timing (sequential vs concurrent), stroke phase, and patient characteristics.^[10]^ Therefore, we conducted a systematic review and meta-analysis of randomized controlled trials (RCTs) to evaluate the efficacy and safety of MT combined with tDCS for improving upper limb motor function and ADL in patients with stroke, and to provide evidence to inform clinical protocol optimization.

## 2. Methods

### 2.1. Protocol registration and reporting

This systematic review and meta-analysis was performed in accordance with the Preferred Reporting Items for Systematic Reviews and Meta-Analyses statement. The review protocol was not prospectively registered in PROSPERO or another public registry. This absence of protocol registration was acknowledged as a methodological limitation.

### 2.2. Search strategy

This study was approved by the Ethics Committee of Zibo Central Hospital. We systematically searched major English databases (PubMed, Embase, Web of Science, and the Cochrane Library) and major Chinese databases (CNKI, Wanfang, VIP) from inception to Nov 2025. Search terms were developed using a combination of controlled vocabulary (subject headings) and free-text terms. Chinese search terms included “脑卒中,” “镜像疗法,” and “经颅直流电刺激.” English search terms included “stroke,” “MT,” and “tDCS.”

A representative PubMed strategy was:

(stroke OR cerebrovascular accident) AND (MT OR mirror visual feedback) AND (tDCS OR tDCS) AND (upper limb OR arm OR upper extremity OR hand) AND (Fugl-Meyer OR action research arm test OR Box and Block OR Barthel) AND (randomized OR trial).

### 2.3. Eligibility criteria

#### 2.3.1. Inclusion criteria

Studies meeting all of the following criteria were included:

1. Participants were diagnosed with stroke confirmed by magnetic resonance imaging or computed tomography.
2. Participants had unilateral upper limb hemiplegia attributable to stroke.
3. Adults aged ≥18 years.
4. RCTs comparing tDCS combined with MT versus MT alone or tDCS alone.

#### 2.3.2. Exclusion criteria

Studies were excluded if: Participants had intracranial hemorrhage, intracranial hypertension, severe cardiac disease, or an implanted pacemaker; Participants had epilepsy, Parkinson’s disease, or other major neurological disorders; The study was non-randomized, duplicated, lacked extractable outcome data, or was not a full-text original report.

### 2.4. Interventions and comparators

The experimental group received active tDCS combined with MT. Control groups received MT alone or tDCS alone. Co-interventions such as conventional rehabilitation and/or pharmacotherapy were allowed provided they were comparable between groups.

### 2.5. Outcomes

All included studies reported quantitative (continuous) outcome measures, with higher scores indicating better recovery. Primary outcomes: Fugl-Meyer Assessment Upper Limb (Fugl-Meyer Assessment for Upper Extremity [FMA-UE]) score; Modified Barthel Index (MBI) score; Wolf Motor Function Test (WMFT) score.

### 2.6. Study selection and data extraction

All retrieved records were imported into EndNote X9 for de-duplication. Two reviewers independently screened titles/abstracts and subsequently assessed full texts for eligibility. Any disagreement was resolved through discussion, with arbitration by a third reviewer if necessary. Data extraction was performed independently by2reviewers and cross-checked. Extracted variables included: first author, publication year, sample size, participant characteristics (sex, mean age, mean disease duration/time since stroke), intervention details (tDCS + MT protocols), and outcome measures. A third investigator verified the overall process for accuracy and completeness.

### 2.7. Risk of bias assessment

The risk of bias of included RCTs was evaluated independently by 2 reviewers using the Cochrane risk-of-bias tool, which assesses: random sequence generation, allocation concealment, blinding, completeness of outcome data, selective reporting, and other potential biases. Each domain was rated as “low risk,” “high risk,” or “unclear risk” of bias, and an overall methodological judgment was summarized for each study.

### 2.8. Statistical analysis

Meta-analysis was performed using Review Manager (RevMan) version 5.4. Heterogeneity was assessed using the I-squared statistic *(I*^2^) statistic and Cochran *Q* test. A fixed-effects model was used when heterogeneity was low (*I*^2^ < 50% and *P* > .10); otherwise, a random-effects model was applied. Because all outcomes within each synthesis were measured using the same scale, effect sizes were expressed as mean differences (MDs) with 95% confidence intervals (CIs). Statistical significance was defined as *P* < .05. Publication bias was not formally assessed with funnel plots or Egger regression because no individual pooled comparison included 10 or more studies, making such tests underpowered and potentially misleading.

## 3. Results

### 3.1. Study selection

**Figure 1.**
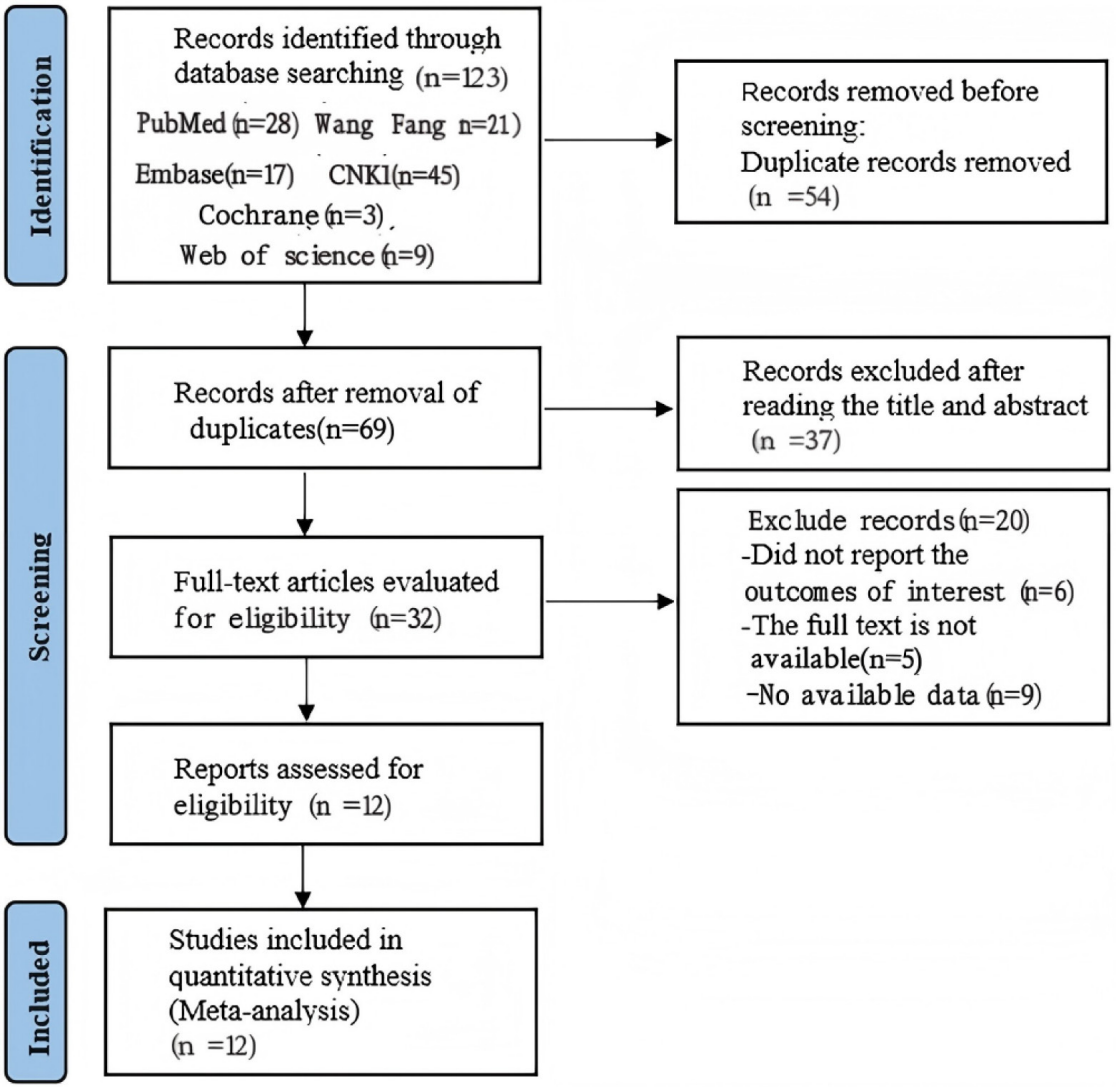
PRISMA flow diagram.

Following Preferred Reporting Items for Systematic Reviews and Meta-Analyses guidelines, database searches identified 123 potentially relevant records. After removing 54 duplicates, 69 records were screened; 37 were excluded; 32 full-text assessed. Of these 32 full-text articles, 20 were excluded because they did not meet the inclusion criteria, lacked extractable data, or used inconsistent interventions. Leading to 12 studies for qualitative synthesis and quantitative meta-analysis^[11–22]^(Fig. 1).

### 3.2. Study characteristics

Included studies were published between 2015 and 2025, enrolling 1024 participants (Table 1). The evidence base comprised 12 studies, all of which evaluated the therapeutic effect of tDCS combined with MT for upper limb motor dysfunction after stroke. The treatment duration varied across trials, with 1 study of short-term intervention, 1 study of medium-term intervention, 8 studies of standard-term intervention, and 2 studies of longer-term intervention. The investigated interventions were consistent in the core combination of tDCS and MT, with the experimental groups receiving conventional rehabilitation training plus tDCS and MT, and the control groups receiving conventional rehabilitation training plus either single tDCS, single MT, or sham tDCS.

**Table 1:** Characteristics of included studies.

Study	Sample size (T/C)	Age (T/C)	Intervention (T/C)	Duration	Period	Outcome
Liao WW2020^[11]^	12/8	–	tDCS + MT/MT	T:20 min tDCS + 20 min MT/C:20 min MT + 30 minCRT)	4w	FMA-UE
Liu Y2023^[12]^	47/46	58.83 ± 5.89/58.76 ± 5.62	tDCS + MT/tDCS	T:tDCS 20 min + 30 min MT/C:20 min tDCS	4w	FMA-UE、Brotez、Barthel
Chen H2020^[13]^	26/26	59.32 ± 8.59/61.31 ± 9.13	tDCS + MT/tDCS	T: 180 min CRT + 50 min MT/C:180 min CRT+ 20 min tDCS	4w	FMA-UE、MBI
Xu P2025^[14]^	15/15	66.67 ± 8.77/62.20 ± 8.88	tDCS + MT/tDCS	T:CRT + 20 min tDCS + 20 min MT/C: CRT + 20 min tDCS	10 d	FMA-UE、MBI
Gao Z2023^[15]^	71/71	71.18 ± 5.13/71.17 ± 5.15	tDCS + MT/tDCS	T: 120 min CRT + 20 min tDCS + 30 min MT/C: 120 min CRT + 20 min tDCS	6 wk	FMA-UE、Barthel、WMFT
Chen Y2022^[16]^	55/55	62.03 ± 8.45/62.84 ± 8.53	tDCS + MT/MT	T: 40 min CRT + 20 min tDCS + 20 min MT/C: CRT 40 min + 20 min MT	4 wk	FMA-UE、MBI
Ren SS2021^[17]^	34/34	61.12 ± 9.98/59.26 ± 11.13	tDCS + MT/ST tDCS	T: 180 min CRT + 20 min tDCS + 30 min MT/C: 180 min CRT + 20 min ST tDCS	4 wk	FMA-UE、MBl、WMFT
Jia PP2025^[18]^	46/46	62.50 ± 8.73/ 61.94 ± 9.25	tDCS + MT/tDCS	T: 60 min CRT + 20 min tDCS + 30 min MT/C: 60 min CRT + 20 min tDCS	4 wk	FMA-UE、MBl、WMFT
Li XL2021^[19]^	162/162	50.37 ± 14.03/52.43 ± 15.12	tDCS + MT/tDCS	T:40 min CRT + 20 min tDCS + 20 min MT/C: 40 min CRT + 20 min tDCS	4 wk	FMA-UE、MBI、ARAT
Zhu L2022^[20]^	18/18	52.11 ± 12.13/53.00 ± 9.74	tDCS + MT/MT	T: CRT + 20 min tDCS + 40 min MT/C: CRT + 60 minMT	4 wk	FMA-UE、MBI、ARAT
Luo YY2025^[21]^	15/15	59.00/62.00	tDCS + MT/MT	T: CRT + 20 mintDCS + 20 min MT/C: CRT + 20 minMT	3 wk	FMA-UE
Cho 20 15^[22]^	14/13	58.29 ± 10.67/60.38 ± 10.19	MT + tDCS/tDCS	T: 20 mintDCS + 20 min MT/C: 20 mintDCS	6 wk	FMA-UE

ARAT = action research arm test, CRT = conventional rehabilitation training, FMA-UE = Fugl-Meyer assessment for upper extremity, MBI = Modified Barthel Index, MT = mirror therapy, ST = sham treatment, tDCS = transcranial direct current stimulation, WMFT = Wolf Motor Function Test.

Time unit abbreviations: min = minute, d = day, wk = week.

### 3.3. Risk of bias

**Figure 2.**
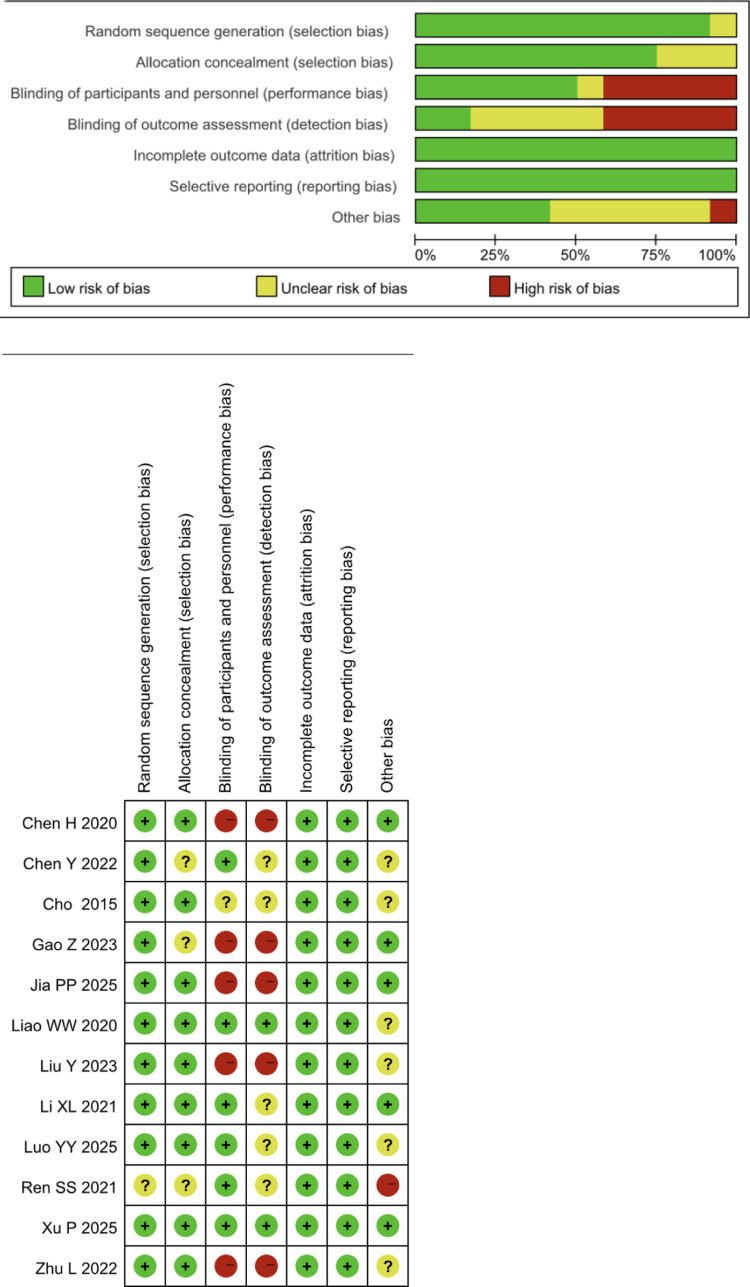
Risk of bias summary.

Risk of bias (assessed via Cochrane Tool 2.0) is summarized in the plots: Most domains (random sequence generation, allocation concealment, incomplete outcome data, selective reporting) showed low bias risk across 12 trials, but blinding (participants/personnel, outcome assessment) had substantial high/unclear risk (due to intervention sensory characteristics). Other biases had minor unclear/high risk. Overall, the trials were judged to be of moderate quality(Fig. 2).

### 3.4. Meta-analysis results

#### 3.4.1. FMA-UE score

**Figure 3.**
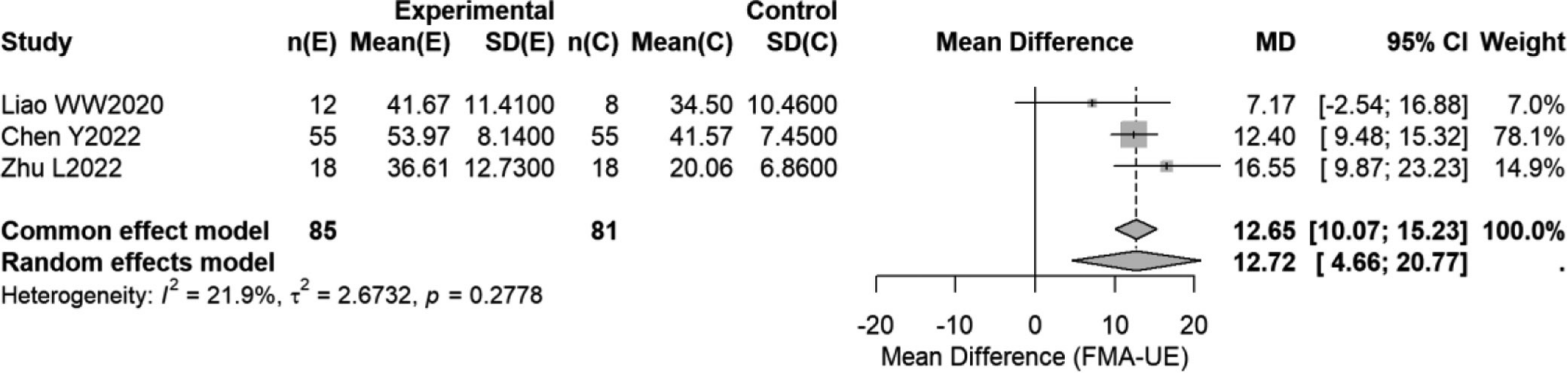
Forest plot of FMA-UE scores in the combined group versus the MT group. CI = confidence interval, FMA-UE = Fugl-Meyer Assessment for Upper Extremity, MD = mean difference, MT = mirror therapy.

Three studies reported FMA-UE comparing the combined intervention group with the MT alone group. Low between-study heterogeneity was observed (*P* = .2778, *I*^2^ = 21.9%); therefore, a fixed-effect model was used. The pooled analysis showed that FMA-UE scores were significantly higher in the combined intervention group than in the MT group (MD = 12.65, 95% CI 10.07–15.23, *P* < .05), indicating that adding tDCS to MT confers additional improvement in poststroke upper limb motor function (Fig. 3).

**Figure 4.**
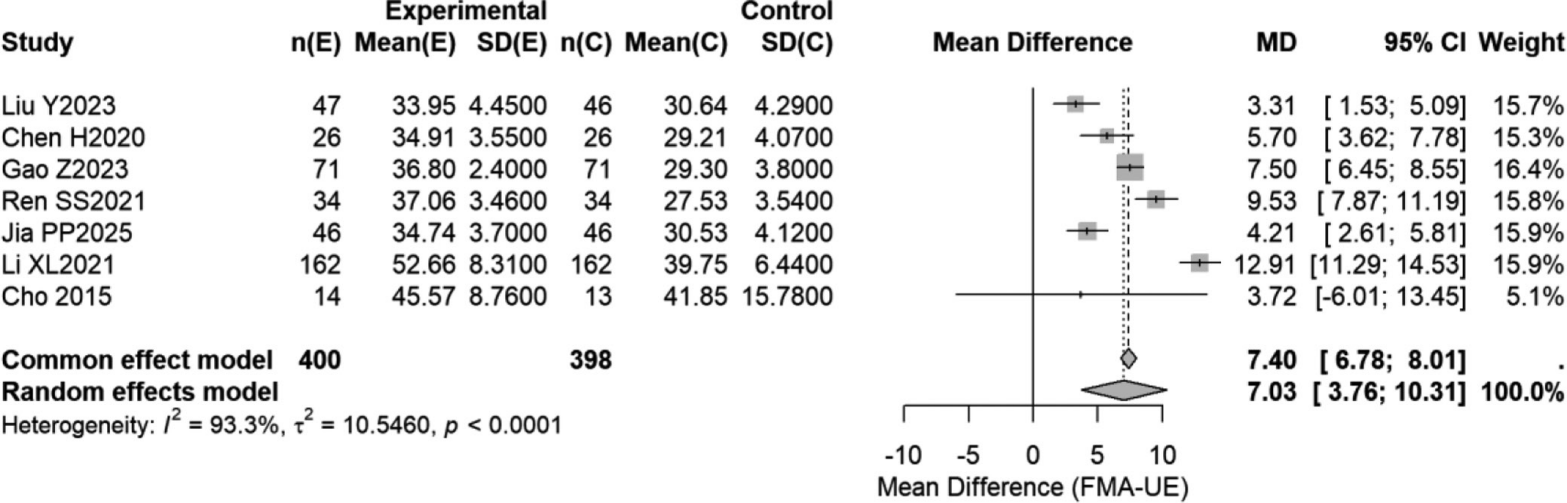
Forest plot of FMA-UE scores in the combined group versus the tDCS group. CI = confidence interval, FMA-UE = Fugl-Meyer Assessment for Upper Extremity, MD = mean difference, tDCS = transcranial direct current stimulation.

Seven studies compared the combined intervention group with the tDCS alone group. Substantial heterogeneity was detected (*P* < .0001, *I*^2^ = 93.3%); thus, a random-effects model was applied. The pooled results demonstrated significantly higher FMA-UE scores in the combined intervention group than in the tDCS group (MD = 7.03, 95% CI 3.76–10.31, *P* < .05), suggesting that MT combined with tDCS is more effective than tDCS alone for improving upper limb motor recovery after stroke (Fig. 4).

#### 3.4.2. MBI score

**Figure 5.**
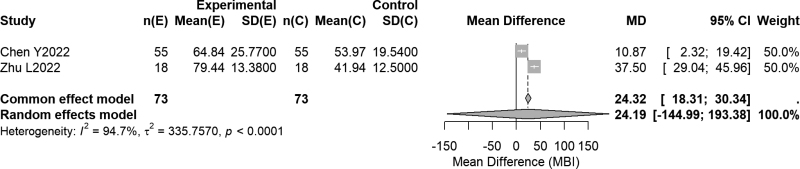
Forest plot of MBI scores in the combined group versus the MT group. CI = confidence interval, MD = mean difference, MBI = Modified Barthel Index, MT = mirror therapy.

Two studies reported MBI scores comparing MT + tDCS with MT alone. Considerable heterogeneity was observed $I{\;}^{2}=94.7$; therefore, a random-effects model was applied. The pooled estimate was highly imprecise and not statistically significant because the 95% CI crossed zero (MD = 24.19, 95% CI: −144.99–193.38, *P* > .05; Figure 5). Therefore, no firm conclusion can be drawn regarding the additional benefit of MT + tDCS over MT alone for ADL.

**Figure 6.**
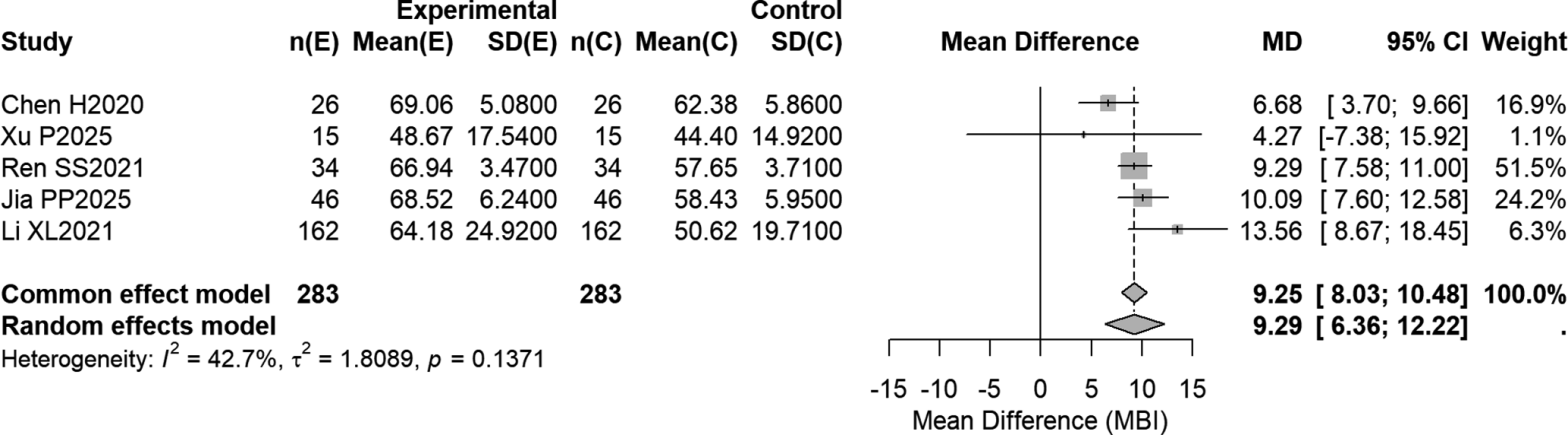
Forest plot of MBI scores in the combined group versus the tDCS group. CI = confidence interval, MBI = Modified Barthel Index, MD = mean difference, tDCS = transcranial direct current stimulation.

Five studies compared MT + tDCS with tDCS alone. Moderate heterogeneity was observed. The pooled results showed higher MBI scores in the MT + tDCS group than in the tDCS alone group (MD = 9.29, 95% CI: 6.36 to 12.22, *P* < .05; Figure 6), suggesting possible improvement in ADL in this comparator subgroup.

#### 3.4.3. WMFT score

**Figure 7.**
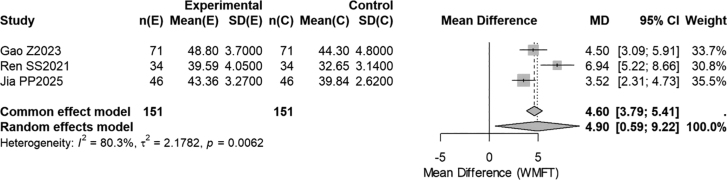
Forest plot of WMFT scores in the combined group versus the tDCS group. CI = confidence interval, MD = mean difference, tDCS = transcranial direct current stimulation, WMFT = Wolf Motor Function Test.

Three studies reported hand function assessed by the WMFT comparing the combined intervention group with the tDCS alone group. Substantial heterogeneity was observed (*P* = .0062, *I*^2^ = 80.3%); therefore, a random-effects model was applied. The pooled analysis showed that WMFT scores were significantly higher in the combined intervention group than in the tDCS group (MD = 4.90, 95% CI 0.59–9.22, *P* < .05), indicating that the combined intervention may provide additional improvement in hand function after stroke (Fig. 7).

## 4. Discussion

### 4.1. Principal findings

This meta-analysis synthesized evidence from RCTs evaluating MT combined with tDCS for poststroke upper limb rehabilitation. Overall, MT + tDCS was associated with improvement in FMA-UE compared with MT alone or tDCS alone. The FMA-UE comparison versus MT alone showed low heterogeneity, whereas the comparison versus tDCS alone remained statistically significant but showed substantial heterogeneity. These findings suggest that the combined intervention may improve impairment-level motor recovery, but the magnitude of benefit varies across protocols and populations.

For functional outcomes, MT + tDCS improved MBI compared with tDCS alone, but comparison versus MT alone was based on only 2 studies and was highly heterogeneous, imprecise, and statistically nonsignificant. Therefore, evidence for improvement in daily activities is less certain than evidence for FMA-UE improvement. WMFT also favored MT + tDCS compared with tDCS alone, although heterogeneity was high. These findings support the potential of combined therapy while underscoring the need for cautious interpretation.

### 4.2. Statistical versus clinical significance

Several pooled effects reached statistical significance; however, statistical significance does not necessarily indicate clinically meaningful improvement. Clinical relevance may depend on baseline severity, stroke phase, intervention duration, patient goals, and minimal clinically important differences for each outcome scale. Therefore, although FMA-UE and WMFT improvements suggest functional potential, the clinical importance of pooled MDs should be interpreted alongside patient-centered outcomes, ADLs, and long-term follow-up.

### 4.3. Plausible mechanisms

The observed benefits are biologically plausible within a neuroplasticity framework. MT provides mirror visual feedback that strengthens action observation–execution coupling and may increase engagement of sensorimotor networks during task practice.^[23–25]^ Meanwhile, tDCS modulates resting membrane potential and cortical excitability, which can facilitate synaptic efficacy and learning-related plasticity.^[26]^ From a “priming + practice” perspective, tDCS may increase the readiness of motor networks to respond to training, while MT supplies repeated, salient, goal-directed practice to consolidate use-dependent reorganization.^[27]^ This complementary mechanism could explain why combining MT and tDCS yields larger functional gains than either intervention alone.

### 4.4. Sources of heterogeneity

Substantial heterogeneity was observed in several comparisons. Potential sources include differences in stroke phase, baseline impairment severity, lesion location, intervention duration, timing of tDCS relative to MT, stimulation intensity, montage, electrode size/current density, MT task selection, training frequency, and total dose. Comparator heterogeneity may also contribute because some studies used MT alone, some used tDCS alone, and others used sham/control stimulation plus conventional rehabilitation. Because most pooled comparisons included only a small number of studies, meta-regression or detailed subgroup analysis by these variables was not statistically reliable. Future trials should report these parameters in detail and predefine subgroup analyses.

### 4.5. Clinical implications

From a clinical perspective, the findings support MT + tDCS as a promising adjunct to upper limb rehabilitation, particularly when the goal is to enhance FMA-UE and improve WMFT. The significant improvement in MBI versus tDCS alone further suggests potential functional relevance. However, because daily living outcomes showed inconsistent precision in the MT comparator analysis and substantial heterogeneity in several outcomes, clinicians should interpret effect magnitude cautiously.

If implemented in clinical settings, protocols should be clearly specified, including: timing of stimulation relative to MT, MT dose and task progression, tDCS montage and current density, and standardized outcome assessment using validated scales. Safety monitoring should be maintained, although reported adverse events were generally mild and transient across trials.

### 4.6. Limitations

Several limitations should be acknowledged. First, the review protocol was not prospectively registered, which may increase the risk of selective reporting. Second, several pooled comparisons included few studies, especially MBI versus MT alone, leading to imprecision. Third, substantial heterogeneity was present in multiple analyses and could not be fully explored because of the small number of studies per comparison. Fourth, methodological limitations such as unclear allocation concealment and incomplete blinding may bias effect estimates. Fifth, comparator definitions varied across trials, and several studies were single-center or local-language publications, limiting generalizability. Sixth, publication bias could not be formally assessed because each pooled comparison included fewer than 10 studies. Finally, adverse events and long-term follow-up were incompletely reported, limiting conclusions about safety and durability of benefit.^[28]^

### 4.7. Future directions

Future research should prioritize adequately powered, multicenter RCTs with standardized reporting of MT dose and tDCS parameters, explicit specification of timing, stratification by stroke phase and baseline severity, and longer follow-up with clinically meaningful endpoints and minimal clinically important differences. Incorporating neurophysiological or imaging markers may also help identify responders and optimize individualized protocol selection.

## 5. Conclusion

In summary, MT combined with tDCS may improve upper limb motor impairment after stroke, particularly as reflected by FMA-UE, compared with MT alone or tDCS alone. However, evidence for ADL and hand function remains less certain because of heterogeneity, imprecision, and the small number of studies in several comparisons. Larger, rigorously designed trials with standardized intervention parameters and longer follow-up are needed to confirm these findings and to define optimal candidates and implementation strategies.

## Acknowledgments

The author wishes to express their gratitude to the team for their valuable contributions, help, and insightful discussions throughout the project.

## Author contributions

**Conceptualization:** Beibei Zong, Chun Zhang, Qingsha Zhang.

**Data curation:** Beibei Zong, Chun Zhang, Qingsha Zhang.

**Formal analysis:** Beibei Zong, Chun Zhang, Qingsha Zhang.

**Funding acquisition:** Beibei Zong, Chun Zhang, Qingsha Zhang.

**Investigation:** Qingsha Zhang.

**Writing – original draft:** Qingsha Zhang.

**Writing – review & editing:** Qingsha Zhang.

## References

[1] Chen JL Lam TK Baniña MC Piscitelli D Levin MF. Neuroimaging and kinematic biomarkers of post-stroke upper limb motor impairment. Neuroimage Clin. 2025;48:103854. 40782519 10.1016/j.nicl.2025.103854 PMC12356466

[2] Pike S Lannin NA Cameron L Palit M Schneider E Cusick A. What stroke survivors say about living with upper limb spasticity and how they manage it. Aust Occup Ther J. 2025;72:e70045. 40905064 10.1111/1440-1630.70045 PMC12409601

[3] Rocha CD Carneiro I Torres M Oliveira HP Solteiro Pires EJ Silva MF. Post-stroke upper limb rehabilitation: clinical practices, compensatory movements, assessment, and trends. Prog Biomed Eng (Bristol). 2025;7:4. 10.1088/2516-1091/adeb1e 40698522

[4] Chen S Chen HB Liao QX. Application progress of transcranial direct current stimulation combined with upper limb rehabilitation therapy on upper limb function in stroke patients with hemiplegia. China J Rehabilit Med. 2021;36:1302–6.

[5] Chan NH Ng SSM. Contribution of perceived upper limb function to the participation and activity levels among community-dwelling people with chronic stroke. Ann Rehabil Med. 2025;49:175–86. 40524518 10.5535/arm.240122 PMC12231404

[6] Vlotinou P Tsiptsios D Karatzetzou S. Transcranial direct current stimulation in conjunction with mirror therapy for upper extremity rehabilitation in chronic stroke patients. Maedica (Bucur). 2022;17:169–76. 35733745 10.26574/maedica.2022.17.1.169 PMC9168586

[7] Xu H Song L Ma Y. Effects of different rehabilitation therapies on upper extremity motor function and activities of daily living in hemiplegic patients with stroke: a network meta-analysis. Medicine (Baltim). 2025;104:e45662. 10.1097/MD.0000000000045662 PMC12599750 41204516

[8] Gómez-García N Álvarez-Barrio L Leirós-Rodríguez R et L. Transcranial direct current stimulation for post-stroke dysphagia: a meta-analysis. J Neuroeng Rehabil. 2023;20:165. 38082316 10.1186/s12984-023-01290-w PMC10712182

[9] Zheng K Guo L Liang W Liu P. Comparison of the effects of transcranial direct current stimulation combined with different rehabilitation interventions on motor function in people suffering from stroke-related symptoms: a systematic review and network meta-analysis. Front Neurol. 2025;16:1586685. 40534746 10.3389/fneur.2025.1586685 PMC12173876

[10] Jin M Zhang Z Bai Z Fong KNK. Timing-dependent interaction effects of tDCS with mirror therapy on upper extremity motor recovery in patients with chronic stroke: a randomized controlled pilot study. J Neurol Sci. 2019;405:116436. 31493725 10.1016/j.jns.2019.116436

[11] Liao WW Chiang WC Lin KC. Timing-dependent effects of transcranial direct current stimulation with mirror therapy on daily function and motor control in chronic stroke: a randomized controlled pilot study. J Neuroeng Rehabil. 2020;17:101. 32690032 10.1186/s12984-020-00722-1 PMC7370428

[12] Liu Y Qian S Sun WL. Effects of transcranial direct current stimulation combined with mirror therapy on post-stroke upper limb and hand function recovery. J Harbin Med Univ. 2023;57:575–9.

[13] Chen H Cai Q Xu L. Effects of transcranial direct current stimulation combined with mirror therapy on upper limb motor function in stroke patients. China Rehabilit Theory Pract. 2020;26:301–5.

[14] Xu P Zhang CJ Wang WL. Observation on the efficacy of transcranial direct current stimulation combined with mirror therapy in the treatment of post-stroke shoulder-hand syndrome. China J Rehabilit Med. 2025;40:1314–9.

[15] Gao Z Zhang HH Yang T. Efficacy of transcranial direct current stimulation combined with mirror therapy in treating upper limb motor dysfunction after stroke. Guangxi Med J. 2023;45:2191–5.

[16] Chen Y Sun YY Zhang QY. Efficacy analysis of transcranial direct current stimulation combined with mirror neuron rehabilitation training in the treatment of post-stroke hemiplegia patients. Electronic J Modern Med Health Res. 2022;6:80–3.

[17] Ren SS Wang XJ Chen AL. Effects of transcranial direct current stimulation combined with music mirror therapy on negative emotions and upper limb motor function in stroke patients with hemiplegia. Chin J Phys Med Rehabilit. 2021;43:1003–6.

[18] Jia PP Wang SJ Gao J. Clinical study on mirror therapy combined with transcranial direct current stimulation for upper limb motor dysfunction in stroke patients. J Aerospace Med. 2025;36:1047–9.

[19] Li XL. Effects of mirror neuron movement imitation training combined with transcranial direct current stimulation on upper limb function and daily living ability in post-stroke hemiplegic patients. J Xinjiang Med Univ. 2021;44:713–7.

[20] Zhu L Qu SW Liu L. Effects of anodic transcranial direct current stimulation combined with mirror therapy on upper limb function in stroke patients. China Rehabilit Theory Pract. 2022;28:1247–51.

[21] Luo YY. Efficacy of Mirror Therapy Combined with Transcranial Direct Current Stimulation in Hand Dysfunction After Stroke [D]. Shanxi Medical University, 2020.

[22] Cho HS Cha HG. Effect of mirror therapy with tDCS on functional recovery of the upper extremity of stroke patients. J Phys Ther Sci. 2015;27:1045–7. 25995552 10.1589/jpts.27.1045 PMC4433973

[23] BroderickJP. Revolution in stroke treatment over 50 years and predicting stroke care in 2050. Stroke. 2026;57:275–84.41099129 10.1161/STROKEAHA.125.052583

[24] Tedla JS Sangadala DR Reddy RS Gular K Kakaraparthi VN Asiri F. Transcranial direct current stimulation (tDCS) effects on upper limb motor function in stroke: an overview review of the systematic reviews. Brain Inj. 2023;37:122–33. 36617689 10.1080/02699052.2022.2163289

[25] Liu C Wang W Zhang CQ. Optimizing tDCS parameters for post-stroke upper limb recovery: a systematic review and meta-analysis protocol. Int J Surg Protoc. 2025;29:68–72. 40860207 10.1097/SP9.0000000000000046 PMC12373094

[26] Wang Y Liu H Li P. Transcranial direct current stimulation attenuates cerebral ischemia-reperfusion injury by inhibiting neuronal pyroptosis via Netrin-1. Neurochem Int. 2025;190:106041. 40876786 10.1016/j.neuint.2025.106041

[27] Wójcik M Vlček P Siatkowski I Grünerová-Lippertová M. Effects of a single tDCS with mirror therapy stimulation on hand function in healthy individuals. Front Hum Neurosci. 2025;19:1607022. 40606492 10.3389/fnhum.2025.1607022 PMC12213558

[28] Waqas S Ahmad A Goulardins JB Hassan Z Hanif A Tariq M. Conjunct effects of transcranial direct current stimulation with mirror therapy on motor control and muscle performance in spastic quadriplegic cerebral palsy children: a randomized clinical trial. J Multidiscip Healthc. 2025;18:1195–216. 40035031 10.2147/JMDH.S506784 PMC11874753

